# Greater numbers of passes and shorter possession durations result in increased likelihood of goals in 2010 to 2018 World Cup Champions

**DOI:** 10.1371/journal.pone.0280030

**Published:** 2023-01-06

**Authors:** Tim Taha, Ahmed-Yahya Ali

**Affiliations:** Faculty of Kinesiology and Physical Education, University of Toronto, Toronto, Ontario, Canada; Sport Sciences School of Rio Maior - Politechnic Institute of Santarem, PORTUGAL

## Abstract

Data analysis in football has indicated an increased likelihood of goals with fewer passes within a possession which have resulted in recommendations of fewer passes and more direct play to score goals. These recommendations did not consider where possessions originated and appear to be contradicted by on-field playing tactics by recent championship winning clubs and national teams in elite competition. Therefore, this study examined the influence of number of passes and possession duration on the likelihood of a shot, or a goal scored during possessions originating in the defensive zone. 4465 possessions originating in the defensive zones of the French, German and Spanish Men’s National teams at the 2010 to 2018 World Cups were analyzed. The possessions were analyzed for the length in time of possession (TP^0.3^), the number of passes completed (nPass^0.425^) and the number of defenders in the offensive zone. Each possession was classified whether or not a shot occurred, a goal occurred or the ball was returned back into the defensive zone. Mixed-effects multivariate logistic regression models were utilized to model the log odds of a shot, goal, or went-back occurrence at the end of each possession. The logs odds of a shot decreased by -0.29 (p = 0.036) with each pass (nPass^0.425^) and the log odds of a goal decreased with time of possession (TP^0.3^) by 1.000 (p = 0.014) and increased with number of passes by 0.775 (p = 0.046). The logs odds of the ball being returned to the defensive zone increased with more passes and greater numbers of defensive players while decreasing with a longer possession duration. The results indicate that a greater number of passes had a positive influence on goal scoring while a longer possession duration had a negative effect. The findings suggest that teams with possessions gained in the defensive zone can use a high number of passes in a short period of time can increase their likelihood of scoring goals.

## Introduction

The notational analysis of football has led to furthering the understanding of tactics in football. Early work clearly established that the number of passes completed in a possession show stochastic properties that could be modeled by a negative binomial distribution [1]. This meant that statistical models could be used to mathematically describe events in football [2]. Further notational work established that success in terms of goals varied between possessions depending on where the possession originated. That is, possessions that originated in the defensive third of the pitch had a much lower likelihood of resulting in a goal than those originated in the attacking third of the pitch [3]. This finding suggests that any analysis should separate possessions and their results by location of where the possession was gained on the pitch. The consequence of not separating locations of where possessions are gained would be an underestimation of the effectiveness of possessions gained in the attacking third and an over-estimation of the effectiveness of possessions gained in the defensive third of the pitch.

The modern tactic of high possession offensive play has a statistical basis in analysis of the 1990 and 1994 World Cup Finals by Hughes and Frank [4]. They found similar results to earlier works in terms of passes per possession and the number of goals resulting from those possessions. However, when the number of goals was normalized to 1000 possessions, the pattern changed to one where there was very little difference between few passes and many passes per possession. This finding suggested that efficiency of turning a possession into a goal was similar between varying possession lengths which suggests that using a high possession, frequent passing offense can yield positive results in terms of goal scoring [4].

Simply examining the passes per possession per goal does not consider that a possession gained in the defensive zone would be difficult to convert into a goal without any passes or just a single pass given the distance from the opposing goal and strength and speed limitations of players. As a result, it is necessary to consider where possessions are gained when counting passes per possession.

Several researchers have found that a significant proportion of possessions are gained in the defensive zone [5–8]. This appeared to be the case across different leagues (Spanish La Liga, German Bundesliga, and English Premier League) where Sarmento et al., found that 71% of 1694 possessions originated in the defensive half of the field [9]. Many possessions originating in the defensive zone are not successful in gaining the area of highest scoring probability. However, they do represent many possessions (1202 possessions in Sarmento’s data, for example [9]) and their scoring potential needs to be maximized [7, 9]. Understanding patterns of successful possessions originating in the defensive zone is important in increasing the scoring efficiency of a possession.

High level football play in the late 2000s to early 2010s evolved to incorporate much higher amounts of passing and possession [10]. Teams such as Barcelona and the Spanish Men’s national team played a high possession, high passing game and won numerous championships. Collett’s timely analysis helped better identify some of the underlying reasons for the success of high possession teams with the major conclusion being that those teams were more greatly skilled at passing and shooting [11]. Beyond better skills, better teams have also shown the ability to create space and goal chances both passively and actively [12]. For example, a player can out sprint a defender to move into open space actively or they can stand passively as a defender moves away to cover another player with the ball. Combining these tactics can negatively influence a defense. A defense that is “imbalanced” due to active or passive creation of space by the offence can result in a higher probability of scoring for the offence [8]. Tenga et al., defined imbalanced as having loose pressure, a lack of back-up and cover [8].

Further potentially influencing the balance of a defense is the amount of time that a possession takes to move the ball from defensive zone to the offensive or scoring area. Gonzalez-Rodenas et al., found that, in the English premier league, possessions longer than 10 seconds resulted in a higher likelihood of offensive penetration but not in creating greater scoring chances [13]. This finding was in contradiction of findings in other leagues [7, 8, 14] where longer possessions were more effective. Sarmento et al., found in Europe’s top three leagues that a shorter possession time was more likely to result in success [9]. However, none of these studies looked at the interaction between where the possession was gained and duration of possession and the likelihood of success.

Simply examining the passes per possession that result in a goal does not consider that a possession gained in the defensive zone would be difficult to convert into a goal without any passes or just a single pass given the distance from the opposing goal and strength and speed limitations of players. As a result, it is necessary to consider where possessions are gained when counting passes per possession. Within these defensively gained possessions, it can be imagined that a longer possession duration would allow defenses to be more balanced [8], thereby lowering the likelihood of shots and goals. Following on that logic, more passes in a possession, unless completed in a very limited time, would also result in a longer possession and a decreased likelihood of more shots and goals being scored.

Given the significant proportion of possessions originating in the defensive zone, it is important for success in football to maximize the goal scoring resulting from those possessions. The goal of the study, therefore, was to examine the role of the duration of a possession, the number of passes and the number of opposing defenders within the offensive zone on the likelihood of shots on goal and goals scored within possessions gained in the defensive zone. We hypothesized that a shorter time between gaining a possession and a shot resulting combined with a lower defensive density would lead to a greater likelihood of a shot or goal occurring. In addition, as a possession can also be returned to the defensive zone, we also examined the effects of the duration of a possession, the number of passes and the number of opposing defenders within the offensive zone on the likelihood of the ball being returned to the defensive zone. In this case, we hypothesized that a longer duration of possession, a larger number of passes and greater numbers of defenders in the offensive zone increased the likelihood of the ball being returned to the defensive zone.

To test these hypotheses, we used the games played in the 2010, 2014 and 2018 World Cups by the respective champions Spain, Germany, and France. As these were the most recent World Cups, they represent the most modern trends in play and facilitated easy access to video of the games. The World Cup teams that we utilized were comprised of very high skill players which avoided potential differences in game tactics due to player skill as noted by Collett [11]. Given that this was a natural experiment, there was relative balance between the numbers of games played by the teams in World Cup winning years and in years when they did not win the championship which allowed us to test if there were differences in the likelihoods of shots or scoring between those years.

## Methods

### Data collection

**Fig 1:**
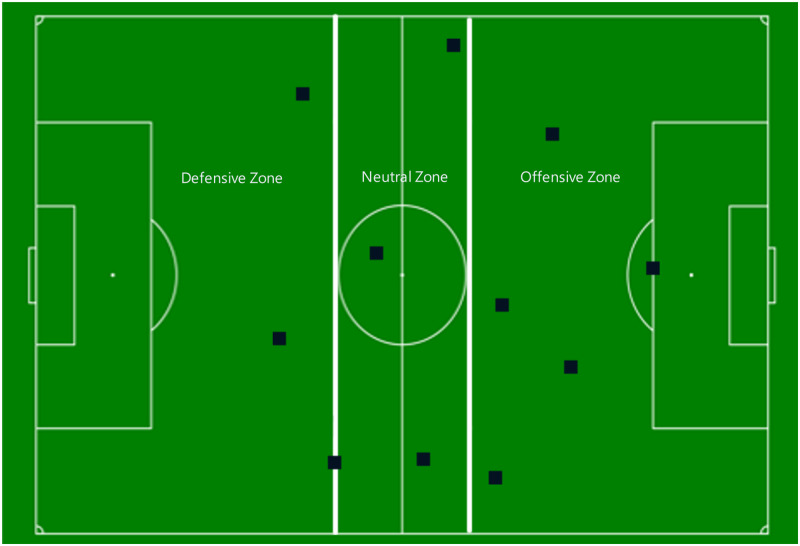
Zones and number of defensive players in the offensive zone. The direction of the attack would be left to right. Black squares represent defensive players. The defensive density in this example would be five.

The forty-six total games of Germany, France and Spain from the 2010, 2014 and 2018 Men’s World Cup Finals were used to conduct the study. Full game videos were viewed in their entirety to collect the data. We were specifically interested in possessions by each of the listed teams that began in their defensive zone and moved into the offensive zone. We defined the defensive zone as being from the team’s own goal line to the centre circle to both sidelines, the offensive zone from the centre circle to the opposing team’s goal to both sidelines (Fig 1). The neutral zone, which is the area to the sidelines from the edges of the centre circle closest to the goals, was included to eliminate uncertainty within the data about whether a possession was gained in either a defensive zone or an offensive zone as it helps give a visual anchor to make a determination of the location of possession gain. The neutral zone ensures that possessions that are counted included movement from a clear defensive zone to a clear offensive zone. Possessions which ended in the neutral zone, or where the beginning or end of the possession could not be seen due to camera angles, were not used in the analysis. Additionally, any possessions where the number of passes could not be clearly identified due to camera angles or players blocking the viewing angle were also excluded. These exclusions resulted in approximately 200 possessions not used in the analysis. In total, Germany had 1684 possessions that fit the criteria and Spain and France each had 1496 and 1285 possessions, respectively.

For each possession, we timed the length of possession (TP), counted the number of passes (nPass) and counted the number of defensive players in the defensive zone (nDef) at the conclusion of the possession. The conclusion of each possession was coded for ending with a shot or not, resulting into a return into the defensive zone while retaining possession or if a goal resulted or not. For example, a possession that ended with a shot but not a goal would be coded “True” for a shot, “False” for returning to the defensive zone and “False” for a goal.

The study was approved by the ethics committee of the Faculty of Kinesiology and Physical Education at the University of Toronto. The committee waived the need for consent. Inter-observer reliability was tested using five randomly selected games. Reliability was good with intraclass coefficients from 0.91 to 0.96.

## Statistical modeling of data

All statistical testing and modeling were completed using the statistical software R [15]. The time of each possession (TP), the number of passes per possession (nPass) and the number of defensive players behind the ball (nDef) were tested for normality using a Shapiro-Wilk test [16]. TP and nPass were found to have a non-normal distribution and were transformed using Tukey’s Ladder of Powers. TP was taken to the power of 0.3 and the nPass was taken to the power of 0.425.

As was noted by Tenga et al., the data lends itself best to using multivariate logistic regression to better understand the complex comparisons [8]. Mixed-effects multivariate logistic regression models were created to model the log odds of a shot, goal, or went-back occurrence at the end of each possession because of the autocorrelation of data within a team during each of the three World Cups. As the outcome variables of a shot, goal or went-back were binomial in nature (i.e., a shot either occurred or did not occur at the end of a possession), a binomial distribution was used. Each model was initially fully saturated with all continuous (TP, nDef, nPass) and factorial (World Cup Champion (T,F), Team(Germany, France, Spain) main effect variables and interactions included [17]. Model simplification was then done by removing non-significant main and interaction effects and re-calculating Akaike’s ‘An Information Criterion’ (AIC) [17]. A combination of minimized AIC and significant main and interactions were used to determine the final models for each of the outcome variables. While the initial fully saturated models had the same structures for each of the three outcomes, the final models for each outcome measure varied after model simplification. The final model for shots took the form:

$$\text{Log}\;\text{odds}\;\text{shots}=\beta_{\text{intercept}}+\beta_{1}\left({\text{TP}}^{0.3}\right)+\beta_{2}\left({\text{nPass}}^{0.425}\right)+\epsilon$$ (1)

$$\beta_{\text{intercept}}=\beta_{\text{mean}\;\text{intercept}}+\beta_{\text{worldcup}|\text{team}\;\text{intercept}}$$ (1a)

where β_1,2_ are the coefficients for the fixed effects TP^0.3^ and nPass^0.425^. The intercepts or random effects were allowed to vary as we felt that there would be an effect of autocorrelation in the data for a given team (France, Germany, Spain) during a given World cup year (2010, 2014 or 2018). ϵ is the error term. The model for goals given a shot is similar:

$$\text{Log}\;\text{odds}\;\text{shots}=\beta_{\text{intercept}}+\beta_{1}\left({\text{TP}}^{0.3}\right)+\beta_{2}\left({\text{nPass}}^{0.425}\right)+\beta_{3}\left(\text{nDef}\right)+\epsilon$$ (2)

$$\beta_{\text{intercept}}=\beta_{\text{mean}\;\text{intercept}}+\beta_{\text{worldcup}|\text{team}\;\text{intercept}}$$ (2a)

Finally, the model for went-back possessions also only had main fixed effects:

$$\text{Log}\;\text{odds}\;\text{went-back}=\beta_{\text{intercept}}+\beta_{1}\left({\text{TP}}^{0.3}\right)+\beta_{2}\left({\text{nPass}}^{0.425}\right)+\beta_{3}\left(\text{nDef}\right)+\epsilon$$ (3)

$$\beta_{\text{intercept}}=\beta_{\text{mean}\;\text{intercept}}+\beta_{\text{worldcup}|\text{team}\;\text{intercept}}$$ (3a)

None of the three final models had overdispersal.

## Results

The study was completed using 46 World Cup finals games with Germany playing 17 games, followed by France with 15 games played and Spain with 14 games played. The total number of possessions starting from the defensive zone and gaining the offensive zone (possessions hereafter) was 4465, with France having 1285, Germany having 1684, and Spain having 1496 possessions. On a per game basis, Spain had the greatest number of possessions per game (106.8) compared with Germany (99.0) and France (85.7). Number of goals scored, and goals given up by each team and each team’s final tournament results are listed in Table 1.

**Table 1:** Games played, number of possessions, GF, GA and outcome of tournament for France, Germany and Spain in the 2010, 2014 and 2018 World Cups.

Team	World Cup	Games played	Possessions	GF	GA	Result
France	2010	3	252	1	4	Group stage
	2014	5	435	7	3	Quarter Finals
	2018	7	598	14	6	Champion
Germany	2010	7	614	16	5	3^rd^ Place
	2014	7	764	18	4	Champion
	2018	3	306	2	4	Group Stage
Spain	2010	7	737	8	2	Champion
	2014	3	254	4	7	Group Stage
	2018	4	505	7	6	Round of 16

GF—Goals For

GA -Goals Against

Model simplification resulted in the dropping of championship winning as a term in all three models. This indicates that the results were valid for teams when they won the World Cup and when they did not win.

### Analysis 1: Possessions with or without a shot

The first analysis completed examined the log odds of a possession resulting in a shot.

As mixed effects modeling was used, both random effects (team by World Cup) and fixed effects (number of passes, time of possession, and number of defensive players) were evaluated. Random effects of team by World Cup year showed variability in intercept log odds of a possession resulting in a shot ranging from a high of -1.81 for France at the 2010 World Cup to –2.28 for Spain at the 2018 World Cup (Table 2). For all teams by World Cup, a greater number of passes (nPass^0.425^) significantly lowered the log odds of a shot resulting from a possession by -0.29 (p = 0.026) (Table 3). For the French team in 2010, for example, a single pass would result in the decrease of the log odds from the intercept value of -1.81 to -2.10 which can be expressed as a change in probability of a possession resulting in a shot from 0.14 to 0.11. With ten passes, for example, the log odds would fall to -2.88 or a probability of 0.07. Time of possession (TD^0.3^) was left in the model as it improved model fit by lowering AIC but did not significantly affect log odds of a shot (p = 0.07).

**Table 2:** Random effect estimated log odds intercepts and base probabilities for possessions that result in a shot for each team at the 2010, 2014 and 2018 World Cup Finals.

World Cup	Team	Estimated Intercept	Probability
2010	France	-1.81	0.14
	Germany	-2.01	0.12
	Spain	-2.11	0.11
2014	France	-1.90	0.13
	Germany	-2.25	0.10
	Spain	-2.25	0.10
2018	France	-2.32	0.09
	Germany	-2.02	0.12
	Spain	-2.29	0.09

**Table 3:** Estimated log odds, standard errors, confidence intervals and p-values for a possession resulting in a shot.

	Estimate	Standard Error	5% Confidence Interval	95% Confidence Interval	p - value
Intercept	-2.11	0.18	-2.46	-1.76	<0.001
Time of Possession (TP^0.3^)	0.24	0.14	-0.03	0.51	0.076
Number of Passes (nPass^0.425^)	-0.29	0.13	-0.54	-0.04	0.026

### Analysis 2: Possessions that resulted in the ball moving back into the defensive zone

The second model examined the log odds of a possession moving the ball back into the defensive zone after gaining the offensive zone.

Of the 4495 possessions, 1051 were taken back into the possessing team’s own defensive zone. With model simplification, no interactions were found. Random effects of team by World Cup year showed variability in intercept log odds of a possession resulting in a shot ranging from a high of -2.68 for Spain at the 2018 World Cup to –5.62 for France at the 2010 World Cup (Table 4). All teams showed the same fixed effect results for each of the World Cups. More passes (nPass^0.425^) and greater numbers of defensive players between the ball and their goal resulted in higher log odds of moving the ball back into the defensive zone (change in log odd nPass^0.425^ = 0.62, p < 0.0001 and change in log odds nDef = 0.12, p = 0.0001). In contrast, the log odds of a longer time of possession decreased (log odds TP^0.3^ = -0.32, p = 0.005) the likelihood of returning the ball back into the defensive zone (Table 5).

**Table 4:** Estimated log odds, standard errors, confidence intervals and p-values for a possession resulting in a return to the defensive zone.

World Cup	Team	Estimated Intercept	Probability
2010	France	-5.62	0.00
	Germany	-3.65	0.03
	Spain	-3.44	0.03
2014	France	-3.64	0.03
	Germany	-2.88	0.05
	Spain	-3.22	0.04
2018	France	-3.30	0.04
	Germany	-2.99	0.05
	Spain	-2.68	0.06

**Table 5:** Random effect estimated log odds intercepts and base probabilities for possessions that result in a return to the defensive zone for each team at the 2010, 2014 and 2018 World Cup Finals.

	Estimate	Standard Error	5% Confidence Interval	95% Confidence Interval	p - value
Intercept	-3.49	0.34	-4.16	-2.82	<0.001
Time of Possession (TP^0.3^)	-0.32	0.11	-0.55	-0.10	0.005
Number of Passes (nPass^0.425^)	0.62	0.12	0.39	0.85	<0.001
Players Behind	0.12	0.01	0.10	0.13	<0.001

### Analysis 3: Possession with a shot that resulted in a goal

The final analysis used the 472 possessions that resulted in a shot. Given a shot occurring at the conclusion of a possession, we examined the log odds of a goal occurring.

Like analyses 1 and 2, there were no interactions. Random effects of team by World Cup showed higher logs odds ranging from 0.03 (Germany 2014) to -1.03 (France 2010) which indicates that the base probability of scoring given a shot for Germany was just over 0.5 at the 2014 World Cup (Table 6). Time of possession (TP^0.3^) had the greatest fixed effect with a decrease in log odds of– 1.000 (p = 0.014) indicating that a longer possession was less likely to result in a goal. The number of passes had a positive fixed effect of 0.775 increase in log odds (p = 0.0464). Greater numbers of defensive players between the ball and their goal resulted in lower log odds of -0.050, but this effect was not significant (p = 0.0502) and was only left in the model as it resulted in an improved model fit (Table 7).

**Table 6:** Estimated log odds, standard errors, confidence intervals and p-values for shots from possessions that originate in the defensive zone that result in a goal.

World Cup	Team	Estimated Intercept	Probability
2010	France	-1.03	0.26
	Germany	-0.15	0.46
	Spain	-0.62	0.35
2014	France	-0.43	0.39
	Germany	0.03	0.51
	Spain	-0.32	0.42
2018	France	-0.27	0.43
	Germany	-0.79	0.31
	Spain	-0.29	0.43

**Table 7:** Random effect estimated log odds intercepts and base probabilities for shots from possessions that originate in the defensive zone that result in a goal for each team at the 2010, 2014 and 2018 World Cup Finals.

	Estimate	Standard Error	5% Confidence Interval	95% Confidence Interval	p - value
Intercept	-0.43	0.53	-1.47	0.61	0.418
Time of Possession (TP^0.3^)	-1.00	0.41	-1.80	-0.20	0.014
Number of Passes (nPass^0.425^)	0.78	0.39	0.01	1.54	0.046
Players Behind	-0.05	0.03	-0.10	0.00	0.050

## Discussion

The aim of the current study was to determine the effects of the number of passes, the duration of the possession and the number of defensive players in the offensive zone on the log odds of a shot, goal or players returning the ball to their own defensive zone in possessions that originate in the defensive zone. As was suggested by Tenga et al., [18] we used multiple logistic regressions to model the data. The log odds of a shot on goal decreased by 0.29 per pass. Given a shot, the log odds of a goal increased by 0.775 with each pass but decreased by 1.0 with each second of the duration of possession. Finally, the log odds of players returning the ball to the defensive zone increased by 0.62 with each pass and by 0.12 with each defender in the offensive zone. These results were common to all teams whether they won the World Cup in a given year or not.

One of the major differences between the present study and previous studies using multiple logistic regression is that we used a mixed-effects approach [5, 7–10, 13, 19–21]. As the different teams during each of the three World Cup Finals would have unique playing characteristics which would introduce pseudo-replication [16] into the data making the team effect dominate the model. By including the teams by World Cup Final year as a random effect, we could better understand the commonalities of the fixed effects (TP, nPass, nDef) amongst all the teams. This approach makes the results potentially more generalizable. Caution must be taken when looking at the values for the random effects of team by World Cup final, as the model was not designed to be able to detect differences in that aspect.

One difficulty in comparing our results in terms of shots and goals with previous studies is that recent literature has not used shots and goals as a measure because, “…goals provide few data points for an entire match and consequently large samples of matches/ team possessions are required for meaningful analysis.” [21] In the present study, we used 46 matches which resulted in 4465 possessions that originated in the defensive zone. Tenga et al., found some similarity in odds ratios between goals scored and score box possessions when comparing long possessions and short possessions [21]. This similarity wasn’t tested statistically. As a result, comparing our findings to those types of studies involves some assumptions of similarity.

Given those assumptions, there are still comparisons to be made between our findings and previous work if we loosely equate shots and goals with a score box possession. Tenga et al., used a measure of pass number which combined duration of a possession with the number of passes [8]. Long possessions (i.e., those with more than 5 passes) had significantly positive odds ratios when compared to short possessions (1 or 2 passes) in gaining the scoring box against un-balanced defenses. This is similar to the finding by Lago-Ballesteros et al., who found that longer possessions had higher log odds of resulting in a score box possession [7]. In contrast, Sarmento et al., found that shots, goals and events such as penalties in top European leagues including the Champions League were more likely with fewer passes and shorter durations [9]. Again, this included possessions gained in the offensive zone, as well as mixing shots, goals and penalties together. Like Sarmento et al., and older studies [3, 4, 9], but in contrast to others [7, 8], we found the log odds of a shot decreased with each pass. The older studies, like the current study, examined international football, unlike the others which studied professional club football. Tactical play may vary between club and international football due to differences in the amount of time training and playing as team. Additionally, the difference in the findings could be attributed to our sample of only defensive zone originating possessions where more passes could be linked to more opportunities for the defensive players to cover spaces where shots are possible.

The finding in the present study that the log odds of a goal given a shot increased with increasing numbers of passes but decreases by the duration of the possession is intriguing. In accordance with our findings, goals have been found to have significantly greater odds ratios with long possessions compared to short possessions when length of possession is determined by number of passes [21]. Adding the time factor, that is the duration of the possession, gives us additional insight about the nature of the characteristics of possessions that are more likely to lead to goals. It is important to note that a shot is only one precondition to a goal. Other preconditions include location of where the shot is taken, the placement of the shot and the positioning of the defenders and goalkeeper relative to the shooter and goal. Our findings suggest that in order to maximize the likelihood of shot becoming a goal, more passes and a shorter duration of possession are necessary. It is possible from our data that players used more quick, short passes in a limited period of time to place the ball into spaces between defenders which allowed better quality shots which became goals. Essentially, teams that we analyzed followed what Collet suggested in that team skill and offensive efficiency was more important than simply possessing the ball or bombarding the goal area with shots [11].

Our final analysis looked at possessions where players after having brought the ball into the offensive zone from their own defensive zone, returned the ball to their own defensive zone. We expected to see that more passes, longer durations of possessions and greater numbers of defenders would increase the log odds of returning to the defensive zone. We were somewhat surprised, then, to find that while the number of passes and greater numbers of defenders had a significant effect, the duration of possession did not influence movement of the ball back into the defensive zone. Additionally, the number of passes had a much larger effect size (0.62) than the number of defenders (0.12). This suggests that players recognize situations unfavourable to making a shot from space that has a high probability of scoring or where further forward progress with the ball may be impeded as the number of passes increase and respond by returning the ball to their own defensive zone. It could be explained that players are using a “reconnaissance in force” approach when attacking. Using a military view, a reconnaissance in force is primarily looking to gain information about defenses and when possible exploit opportunities for tactical success [22]. While any analogy between military operations and football is not perfect, there are some similarities that can help understanding of the intent behind high passing tactics. Players using multiple passes probe the defense for space to exploit. If none is available due to multiple reasons including, as we found, increased defensive players in the offensive zone, a possession may be returned to the attackers’ own defensive zone in the hope that the defense will change during the next probing possession or more direct attack. A direct possession [13] would be less of a reconnaissance and more of a committed attack where if the objective is not fulfilled (e.g., a shot) there is no opportunity to re-group and attempt a different type of attack. It is important to note that two of the teams we used in our sample (Germany, Spain) played a higher possession style game for the most part which may influence the results.

An important limitation to the results of the study stem from the use of teams that won the World Cups finals in 2010, 2014, and 2018. While it could be argued that these were the most successful teams and should be studied as a result, generalizing is more difficult in that case. As Collett noted, what makes tactical sense for elite teams does not necessarily apply to lesser teams [11]. It can also be argued that while each of the teams won the World Cup during the years studied, they were all also eliminated in the first round as well. We included winning the World Cup as a factor in our initial saturated model but eventually it was excluded due to non-significance suggesting that winning did not play a role in determining the likelihood of shots, goals and going back to the defensive zone occurring with a possession originating in the defensive zone. Any conclusions drawn from the current study are also limited by the lack of precision in determining the location and situation at the beginning of the possession. For example, it is conceivable that a possession will have different characteristics whether it is a throw-in from close to the centre circle or a goal kick. Tactical play may also have varied depending on the opponent’s defensive system or the match status. These limitations also represent interesting and important directions in future research but may have to involve different types of data collection and analysis such as spatial analysis. Finally, it should be noted that high possession football was very much in mode during the first part of the 2010s and a similar study done over three different World Cups finals may get varying results [11].

From a practical point of view, the findings suggest that possessions beginning in the defensive zone have some common elements in order to achieve tactical success. In terms of goal scoring, our findings imply that a moving the ball quickly to a shooting position is important. Moving the ball to a shooting position can be accomplished through higher numbers of passes as long as they are completed in a short period of time. This could mean that passes can be short in length but have to be completed quickly moving towards the opponent’s goal to increase the likelihood of scoring. Further analysis looking at whether this is better accomplished through one-touch passing as opposed to multiple touches or dribbling is warranted. In addition, players use the higher passing possessions as reconnaissances in force to probe the defense then fall back to try to open up other tactical opportunities which could involve a direct play.

## Conclusions

In conclusion, in World Cup winning teams, with possessions that originated in the defensive zones, the logs odds of a shot decreased with more passes but in the case of possessions given a shot that resulted in a goal, the log odds of a goal increased with more passes and a shorter duration in time. Finally, with defensive zone originating possessions, players had higher log odds of going back to their own defensive zone with higher numbers of defenders in the defensive zone and more passes.
